# Cancer-associated fibroblasts: The chief architect in the tumor microenvironment

**DOI:** 10.3389/fcell.2023.1089068

**Published:** 2023-01-30

**Authors:** Mrinmoy Sarkar, Tristan Nguyen, Esheksha Gundre, Olajumoke Ogunlusi, Mohanad El-Sobky, Biplab Giri, Tapasree Roy Sarkar

**Affiliations:** ^1^ Department of Biology, Texas A&M University, College Station, TX, United States; ^2^ Cancer Biology and Inflammatory Disorder Division, CSIR-Indian Institute of Chemical Biology, Kolkata, India; ^3^ Department of Physiology, University of Gour Banga, English Bazar, India

**Keywords:** CAF, breast cancer, tumor microenvironment, heterogeneity, targeting

## Abstract

Stromal heterogeneity of tumor microenvironment (TME) plays a crucial role in malignancy and therapeutic resistance. Cancer-associated fibroblasts (CAFs) are one of the major players in tumor stroma. The heterogeneous sources of origin and subsequent impacts of crosstalk with breast cancer cells flaunt serious challenges before current therapies to cure triple-negative breast cancer (TNBC) and other cancers. The positive and reciprocal feedback of CAFs to induce cancer cells dictates their mutual synergy in establishing malignancy. Their substantial role in creating a tumor-promoting niche has reduced the efficacy of several anti-cancer treatments, including radiation, chemotherapy, immunotherapy, and endocrine therapy. Over the years, there has been an emphasis on understanding CAF-induced therapeutic resistance in order to enhance cancer therapy results. CAFs, in the majority of cases, employ crosstalk, stromal management, and other strategies to generate resilience in surrounding tumor cells. This emphasizes the significance of developing novel strategies that target particular tumor-promoting CAF subpopulations, which will improve treatment sensitivity and impede tumor growth. In this review, we discuss the current understanding of the origin and heterogeneity of CAFs, their role in tumor progression, and altering the tumor response to therapeutic agents in breast cancer. In addition, we also discuss the potential and possible approaches for CAF-mediated therapies.

## Introduction 

Tumors are heterogeneous, which is one of the hallmarks of malignancy. The progression of the tumor depends on the dynamic crosstalk between cancer cells and the other cells in the stromal microenvironment. One of the key players in the tumor microenvironment is cancer-associated fibroblasts (CAFs). CAFs promote tumor progression, extracellular matrix (ECM) remodeling, inflammation, chemoresistance, and immunosuppression (Ohlund et al., 2014; Kalluri, 2016; Czekay et al., 2022). The exact origin of different subtypes of CAFs in breast cancer is not fully understood. Tumor-secreted factors are critical in controlling CAF-precursors’ differentiation into CAFs (Houthuijzen and Jonkers, 2018). Due to the heterogeneity, targeting CAFs remains a significant challenge. In this review, we discuss the current information regarding CAFs biology, origin, function, and how targeting CAFs, is being explored as an opportunity to improve cancer therapies.

## Origin of CAFs

Despite being of enormous importance for understanding tumor microenvironment (TME), the lack of precision of the identification markers poses a colossal impediment en route to understanding the source of CAFs. It becomes even more inexplicable when no discernible difference is detectable between the surface markers of normal tissue fibroblasts and CAFs. In order to seal the knowledge gap, several researchers have focused on tracking the fibroblastic properties in the TME at various phases, such as tumorigenic mutation induction and the development of *in situ* and invasive cancer. These investigations’ findings on human tissue showed that the so-called fibroblastic stroma underwent gradual alterations, and the preliminary step-up in their number eventually paved the way for the induction of malignancies. Noteworthy to mention is that the CAFs continue to encircle the pertinent cancerous lesions as they gradually transform during the early and premalignant episodes. Experimental data suggest circumscribing fibroblasts may have a tumor-suppressive role in the early stages (Rhim et al., 2014). Experimental evidence supporting the increase in the number of stromal fibroblasts alongside the progression of tumorigenesis provides us with a new concept of *stromagenesis*. This, in turn, prompts the assumption that fibroblasts have a mesenchymal origin and are activated and converted into CAFs during stromagenesis. The new lineage of fibroblasts plays a pro-tumorigenic role, opposite to the initial tumor suppressive role (Sahai et al., 2020a).

Other than differentiating CAFs from normal fibroblasts (NFs) based on inadequately exclusive cell surface markers, the change in shape and size of the NFs was demonstrated to have happened upon treatment with conditioned cell culture medium from human breast cancer cell lines, and this has been associated with conversion into spindle-shaped CAFs (Kumar et al., 2014). However, the entire concept of cellular changes happening in stromal fibroblasts cannot be addressed longitudinally in human biopsy specimens. Although longitudinal sampling is feasible, direct monitoring of stromal fibroblasts is not. Therefore, efforts have concentrated on animal models with properly labeled cells that can track disease development. This has been accomplished by using tissue-specific expression of the “cre-recombinase” in competent laboratory animals with an irreversibly activated reporter gene in cre-positive cells. It might have been easier in the presence of appropriate fibroblast markers (Ping et al., 2021). The “cre-recombinase” technique might have unraveled the potential origin of CAFs to be pericytes, adipocytes, mesenchymal stem cells (MSCs), and endothelial cells. Yeon et al. (2018) found substantial data in favor of the conversion of endothelial cells to CAFs. Data concludes that melanoma-derived exosomes can induce the dedifferentiation of endothelial cells into MSCs (endothelial to mesenchymal transition; EndMT), which are then turned into CAFs. Karnoub et al. (2007) revealed a similar paradigm that explains the transformation of externally administered bone marrow-derived MSCs into CAFs. An interesting study by Nair et al. (2017) demonstrated the effect of conditioned media from T47D and BT549 human breast cancer cells in converting induced pluripotent stem (iPS) cells first into cancer stem cells (CSCs) and then into CAFs.

In contrast, the transition from adipocytes has also been described (Karnoub et al., 2007; Nieman et al., 2011; Bochet et al., 2013). In other circumstances, CAFs are said to prevent adipocyte differentiation. Adipocytes, on the other hand, offer metabolic support regardless of whether they transform into CAFs (Karnoub et al., 2007; Sahai et al., 2020a; Ping et al., 2021). Evidence-based studies showed that Platelet-derived growth factor-BB (PDGF-BB)/Platelet-derived growth factor receptor β (PDGFRβ) signaling system drives pericyte differentiation toward fibroblasts (Hosaka et al., 2016). Further, the epithelial-to-mesenchymal transition (EMT) also feeds the MSCs to CAFs transformation cascade, implying that CAFs have an epithelial origin (Fiori et al., 2019; Mao et al., 2021; Ping et al., 2021). The cytokine generation by the resultant CAFs accounts for tumor cell EMT and promotes cancer spread and invasion (Fiori et al., 2019; Ping et al., 2021).

Information alludes to the idea that both canonical and non-canonical transforming growth factor beta (TGFβ) pathways have a part in fostering the generation of CAFs (Wu et al., 2021). The propagation and differentiation of NFs were correlated with the transmission of TGF and endocytosis by the NFs (Wu et al., 2021). TGFβ and nuclear factor-κB (NF-κB) signaling pathways regulate the conversion of stromal fibroblasts to CAFs in response to a wide range of stimuli, including osteopontin (OPN), interleukin-1 (IL-1), and others that are part of the secretome of the immunological or cancer cells (Fiori et al., 2019). Furthermore, an altered energy metabolism may be vital in converting NFs to CAFs. In the TME, cancer cell-secreted Lysophosphatidic acid (LPA), TGFβ1, or platelet-derived growth factor (PDGF) can potentially trigger the HIF-1α pathway to cause aerobic glycolysis in the fibroblasts (Wu et al., 2021). The transformation of NFs into CAFs is also associated with alterations in how some components express themselves, as shown in several reports (Calvo et al., 2013; Sahai et al., 2020a; Shen et al., 2020). For instance, Yes-associated protein 1 (YAP1), a transcriptional coactivator in NFs, alters the transcription of proto-oncogene c-Src (*src*) by associating with TEA domain transcription factor-1 (TEAD1) to construct a protein compound, which activates cytoskeletal proteins and leads to the development of CAFs (Calvo et al., 2013; Sahai et al., 2020a).

Physiological and inflammatory stress could also affect NFs to CAFs conversion (Sahai et al., 2020a). IL-1 and IL-6, potent inflammatory cytokines, can predominantly trigger CAF activation *via* the NF-κB and signal transducers and activators of transcription (STAT) transcription factors (Erez et al., 2010; Sanz-Moreno et al., 2011a). Additionally, crosstalk and positive feedback involving Janus kinase (JAK)-STAT signaling, the contractile cytoskeleton, and histone acetylation all contribute to CAF activation (Albrengues et al., 2014; Albrengues et al., 2015). Heat shock factor-1 (HSF-1) is synthesized in response to physiological stress, and it is thought to enhance stromagenesis and malignancy *via* transcriptional modification of CAFs (Scherz-Shouval et al., 2014; Sahai et al., 2020a). Other genomic stressors, such as dsDNA break, can initiate an IL-6-mediated response as well as the production of TGFβ family ligand activin A, resulting in the expansion of CAFs or, in some cases, senescent fibroblasts, the removal of which can have severe repercussions for disease relapse (Scherz-Shouval et al., 2014; Ferrari et al., 2019; Sahai et al., 2020a).

## Heterogeneity and plasticity of CAFs

Several experimental reports suggest that the nature of CAFs and the TME remains highly volatile and diverse depending upon different signaling pathways and the organ in which they are located. For instance, FGF and TGFβ trigger different regulatory networks through different effector genes to promote carcinogenesis or regulate immune surveillance (Bordignon et al., 2019; Ping et al., 2021). Induction by TGFβ opens up a cascade of events that stimulates tumor cell invasion beyond basal lamina and EMT (Tsubakihara and Moustakas, 2018). As cancer progresses, the alteration of signaling pathways involved in CAF transition from a tumor-suppressive state to a tumor-promoting activated state shows the transcriptomic significance of CAF phenotype plasticity (Ping et al., 2021).

The vast number of activities ascribed to CAFs in myriad experimental models raises the topic of whether a particular kind of CAF accomplishes all of these functions simultaneously or whether CAFs subspecialize or switch between diverse functional states. Enumerable data confirm CAF specialization, which may be similar to the specialization of NFs. The terms “myofibroblast CAFs or myCAFs” and “inflammatory CAFs or iCAFs” are frequently used to refer to different CAFs with the prefixes implying a myofibroblast phenotype and the regulation of inflammatory response, respectively (Öhlund et al., 2017; Biffi et al., 2019). The CAFs nearest in proximity to the cancerous cells in pancreatic cancer, display a myCAF-like phenotype, which is characterized by strong TGFβ-driven αSMA expression and a contractile nature (Öhlund et al., 2017; Sahai et al., 2020a). iCAFs are marked as having elevated IL-6 expression patterns. The TGFβ-mediated regulation of the IL-1 receptor, which is responsible for initiating NF-κB signaling and subsequent IL-6 production, can be used to explain the apparent exclusivity of the two phenotypes (Biffi et al., 2019; Sahai et al., 2020a; Ping et al., 2021).

Bartoschek et al. (2018) categorized breast CAFs into four subpopulations according to vascular development (vCAF), ECM-enriched signaling (mCAF), expression of cycling or proliferative phases (cCAF), and variously expressed differentiation genes (dCAF), *via* single-cell RNA-sequencing. Another perspective is the assortment of CAF populations based on their origin and location within the TME. vCAFs include angiogenic and vascular regulation functions while predominantly localized near the supposed vasculature. The source of mCAFs was concluded to be of similar marker expression with resident fibroblasts in local mammary tissues. The cCAFs were demonstrated to share the same cluster as vCAFs, being distinguishable only by their proliferating nature. The dCAFs share expression patterns with various stem cells suitable for their putative role in cellular differentiation and tissue morphogenesis (Bartoschek et al., 2018). The demonstrated spatial distribution heterogeneity in these subpopulations elucidates the importance of CAF function concerning their origin and function in inducing a tumor-promoting milieu.

Similarly, Friedman et al. (2020) identified two primary subpopulations of CAFs in breast cancer: pCAFs and sCAFs, which are distinguished by the expression of podoplanin (Pdpn) and S100A4 (also called fibroblast-specific protein one; FSP1), respectively. The makeup and function of these CAFs change as cancer advances and the ratio of different populations give insight into how the patient may respond (Friedman et al., 2020; Asif et al., 2021). The CAF subpopulations classified have distinct phenotypical heterogeneity that distinguishes them from each other. pCAFs were separated into six subgroups based on varying transcriptional differences involving both early and late immune regulation, immune-regulatory signatures, ECM modulation, and inflammation (Friedman et al., 2020). Over time, the transcriptional signatures in breast cancer CAFs transitioned from immune regulatory functions to wound healing and antigen-presentation, as seen with the fraction of pCAFs decreasing over a 4-week period after tumor injection (Friedman et al., 2020). The dynamic nature of CAFs and their ability to transdifferentiate *via* reprogramming have been further demonstrated through lineage analysis showing the origins of pCAFs from NFs and sCAFs from bone marrow-derived MSCs (Friedman et al., 2020).

**FIGURE 1:**
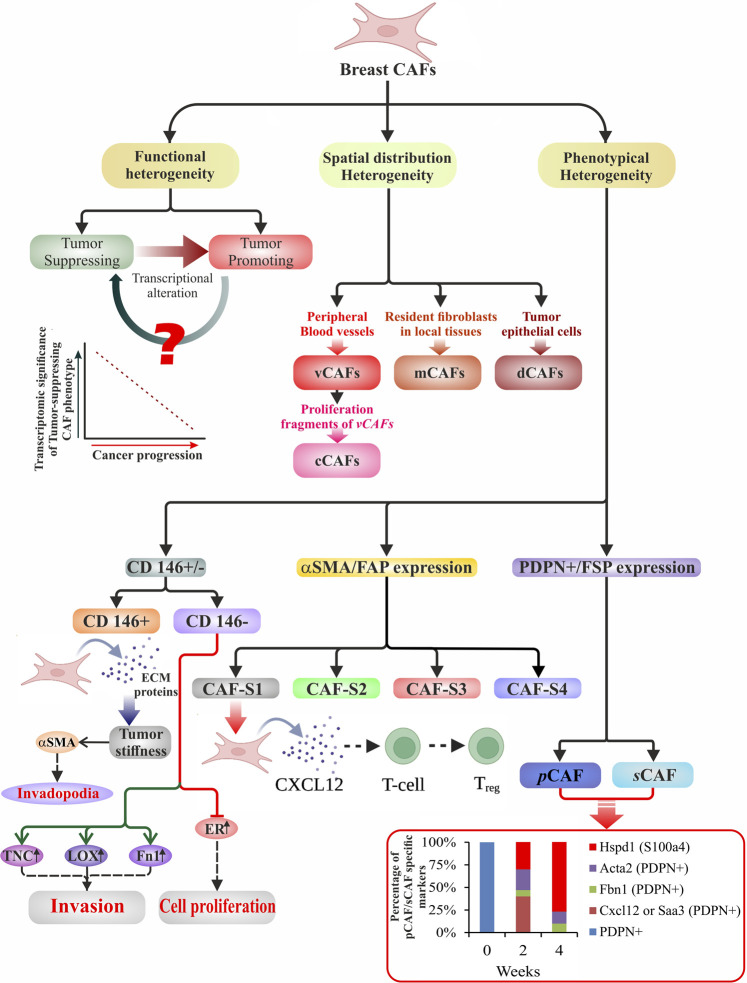
Schematic illustration of heterogeneity in breast CAFs. Broadly, breast CAFs are distinguished under three subclasses based on their heterogeneous function, spatial distribution, and cell surface phenotype. They have been divided into tumor-promoting and tumor-suppressing cell types due to their stage-specific distinct approach toward tumor cells. With the progression of cancer, transcriptional alteration of the tumor-suppressing CAF phenotypes results in the generation of tumor-promoting breast CAFs. Manipulation of the event may prove to be a potent therapeutic tool. Bartoschek et al. (2018) used single-cell RNA data to classify the CAFs according to their spatial distribution. Vascular development, ECM-enriched signaling, expression of proliferative genes, and variously expressed differentiation genes shaped their classification into vCAFs, mCAFs, cCAFs, and dCAFs. Phenotypically breast CAFs could be sub-classified depending upon the i) presence or absence of CD146 (Brechbuhl et al., 2020), ii) expression of αSMA/FAP (Costa et al., 2018), and iii) expression of PDPN/FSP (Friedman et al., 2020). This diagram displays the several subtypes with phenotypical distinctions that are accountable for classified functions at various phases of carcinogenesis. *The bottom right graph* illustrates the percentage of pCAF/sCAF specific markers over a period of 4 weeks in a breast cancer model.

Likewise, other diverse types of CAF phenotypes have been identified in breast cancer, with fibroblast activation protein (FAP) being the primary discriminating marker (Costa et al., 2018). Experimental demonstration correlated an elevated expression of FAP in FAP^+^ fibroblasts with regulatory T-cell (T_reg_) dependent immunosuppression and poor prognosis. Nonetheless, the FAP^+^ targeted chimeric antigen receptor (CAR) T-cell therapy unraveled its role in matrix formation besides being considered a potent immunomodulator (Lo et al., 2015). FAP^+^ fibroblasts have also been identified in PDAC as tumor-promoting CAFs, performing possibly through stromal-derived factor 1 (SDF-1) and C-C motif chemokine ligand 2 (CCL2) dependent manner (Yang et al., 2016a; McAndrews et al., 2022). At the same time, in a genetically engineered mouse model system for PDAC, αSMA^+^ cells were also identified as a type of myofibroblasts (myCAFs) (Djurec et al., 2018; Sperb et al., 2020), believed to be tumor-restraining in nature. McAndrews *et al.* (2022) (McAndrews et al., 2022) used a single cell RNA-seq (scRNAsq) analysis that revealed a minimal overlap between FAP^+^ and αSMA^+^ type CAFs deciphering them as distinct populations with distinct effects on immune response, chemotherapeutic agents, and chemokine secretion. Costa *et al.* (2018) (Costa et al., 2018) defined populations of breast CAFs based on the expression of FAP, αSMA, and other various markers such as FSP1, CD29, PDGFR-β, and caveolin-1 (CAV1). CAF-S1 is seen as having a higher expression of all six markers than CAF-S2, which is negative for all. CAF-S3 involved CD29, FSP1, and PDGFR-β expression, and the CAF-S4 subpopulation consisted of high αSMA and CD29 expression along with low to medium expression of FSP1 and PDGFR-β and negative to low expression of CAV1. However, CAF-S1 and CAF-S4 subsets secrete TGFβ and CXCL12 to activate NOTCH signaling and have been associated with TNBC and HER2^+^ breast cancers, respectively. Along with phenotypical differences, CAF-S1 regulates T-cells, controlling the immune response and suppression in the TME *via* secretion of IL-6/17/10 and CXCL12 (Costa et al., 2018). Understanding the dynamics of CAF transitions and origins with respect to their differing subpopulations can lead to the precise targeting of tumor-promoting CAF subpopulations (Figure 1).

## Functions of CAFs

As documented in the previous section, multiple origins of CAFs account for their equally diverse nature of functions in the TME. They are a significant source of secretory molecules such as growth factors (GFs), cytokines and chemokines, extracellular vesicles (EVs) like exosomes, and extracellular matrices (ECMs). Therefore, the cellular contribution of the CAFs could potentially aid in the initiation and progression of cancer. It also aids in regulating therapeutic resistance through immunomodulation, inflections of local tissue metabolisms, and regulation of the mechanisms establishing hallmarks of cancer, culminating in invasion and metastasis.

However, based on functions, the CAFs may be categorized into two significant subclasses; tumor-promoting CAFs and tumor-suppressing CAFs. Their extraordinary capacity to restructure tumor blood vessels and the extracellular matrix accounts for their reciprocating roles in the TME (Sewell-Loftin et al., 2017). Even though CAFs appear to prevent cancer initially, research findings (Benyahia et al., 2017; Sewell-Loftin et al., 2017; Shen et al., 2017) indicate that they do stimulate cell growth. By forming a “restrictive barrier” encircling a lesion, CAFs effectively prevent the spread of cancer cells by force-mediated ECM-remodeling and through the production of matrix-crosslinking proteins, thus rendering the tumor tissue stiff (Kechagia et al., 2019; Sahai et al., 2020a; Zeltz et al., 2020a; Ping et al., 2021). The altered ECM aids the tumor in developing a therapeutic resistance barrier and a protective canopy to prevent immune cell penetration inside the TME as the disease progresses and the TME evolves (Ping et al., 2021). Apart from promoting metastasis, *de novo* stimulation of fibroblasts stimulates the release of periostin (POSTN) and tenascin-like molecules, which stimulate Wnt signaling (Chiquet-Ehrismann et al., 2014; Yu et al., 2018a). CAFs thereby accomplish the embodiment of macro-metastasis by regulating stem cell niches through Wnt signaling (Oskarsson et al., 2011; Calon et al., 2012; Malanchi et al., 2012; Dumont et al., 2013a; Madsen et al., 2015; Nabhan et al., 2018). Changes in ECM architecture influence the recruitment of infiltrating leukocytes, which has relevance for tumor immune surveillance (Kaur et al., 2019).

Other secretory functions of CAFs, such as vascular endothelial growth factor (VEGF) synthesis by the stromal cells, can lead to angiogenesis (O'Connell et al., 2011; Fukumura et al., 1998). Various cyto- and chemokines affect the immune response by acting upon CD8^+^ T-cells, T_reg_ cells, and macrophages (Monteran and Erez, 2019a). Nevertheless, it is widely agreed that the putative function of CAFs is immunosuppressive, with CXC-chemokine ligand 9 (CXCL9) and TGFβ all playing well-established roles in limiting T-cell responses (Fearon, 2014). Antigen cross-presentation by CAFs has been identified more recently (Elyada et al., 2019); this may cause CD4^+^ T-cell activation and CD8^+^ T-cell suppression (Lakins et al., 2018a). Further evidence from clinical studies points to an antagonistic relationship between CAFs and CD8^+^ T-cells (Costa et al., 2018).

**FIGURE 2:**
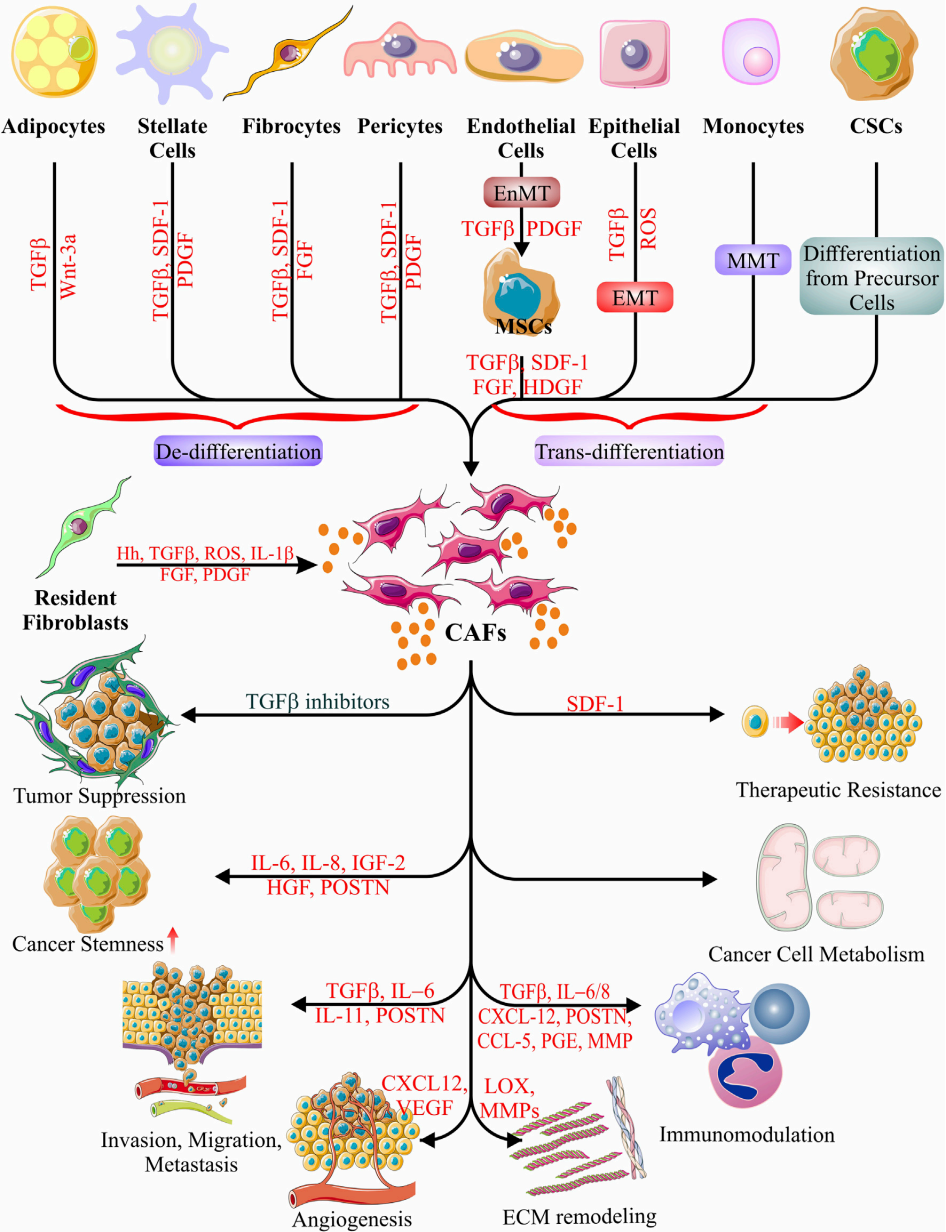
Origin and function of CAFs. Schematic representations of possible CAF cell origins have been depicted, as evidenced to date. Different types of cellular differentiation, trans-differentiation, and de-differentiations give rise to the generation of CAFs. CAFs show a plethora of activities in accord with and against cancer cell survival and malignancy, evidently in a stage-specific manner. The only event that results in the suppression of tumors occurs when the CAF-derived secretome includes TGFβ inhibitors. The other roles of CAFs in cancer include the induction of all the hallmarks of cancer *via* the secretion of various cytokines, chemokines, growth factors, carbohydrate intermediates, and nitrogen sources (amino acids). Their activities include induction of cancer stemness, metastasis, migration, invasion, angiogenesis, ECM remodeling, immunomodulation, metabolic manipulation, and therapeutic resistance (Karnoub et al., 2007; Mani et al., 2008; Erez et al., 2010; Baccelli and Trumpp, 2012; Oskarsson et al., 2014; Albrengues et al., 2015; Yeon et al., 2018; Fiori et al., 2019; Sahai et al., 2020a; Shen et al., 2020; Loh and Ma, 2021; Ping et al., 2021; Wu et al., 2021).

Additionally, IL-6 may facilitate immunosuppression by altering metabolism systemically (Flint et al., 2016). In cancer cells, targeting focal adhesion kinase (FAK) reduces stromal fibroblast activation and the emergence of an immunosuppressive milieu. Interfering with the activity of CXCL12 generated by CAFs enhances T-cell-mediated tumor control (Kraman et al., 2010a; Feig et al., 2013; Chen et al., 2019). TNFα can stimulate fibroblast activation in some circumstances, but it is also known to be suppressed by the tumor-promoting immunosuppressive activities of FAP^+^ fibroblasts (Kraman et al., 2010a; Chaudhry et al., 2013; Lau et al., 2017). Tumor-promoting immunosuppressive activity of FAP^+^ fibroblasts is connected with the inhibition of TNFα signaling. However, in some circumstances, TNFα can also stimulate fibroblast recruitment, making the situation with tumor necrosis factor (TNFα) generated by CAFs more complicated (Kraman et al., 2010b; Chaudhry et al., 2013; Jiang et al., 2016; Lau et al., 2017). The schematic representation explains the diverse origins and multiple functions of CAFs in Figure 2.

Based on a recent study that revealed matrix-specific hedgehog signaling inhibits tumor growth and invasion by lowering matrix stiffness, scientists have focused on the role of matrix conversion in switching pro-tumor CAFs to anti-tumor CAFs (Yoshida, 2020). In smooth muscle cells and allied CAFs (αSMA^+^ (αsmooth muscle actin) CAFs), it has been found that an upsurge in hedgehog signaling is correlated with a proportionate suppression of malignant development (Yang et al., 2016b).

## Role of CAFs in cancer

### Role of CAFs in *stemness* of cancer

The prevalence of CAFs throughout the malignant transformation, from early to late stages, implies that they play an indispensable role in tumorigenesis and progression (Erez et al., 2010). The heterogeneity data demonstrates that the proximal CAFs are empowered with potent juxtacrine TGFβ signaling those aids in transformation into stem cells (Bellomo et al., 2016; Fiori et al., 2019; Nakano et al., 2019; Sahai et al., 2020a). Additionally, the neoplastic cells educate the CAFs to become able to assist through tumor progression and dissemination in other parts of the body (Sahai et al., 2020a; Loh and Ma, 2021; Ping et al., 2021).

Hypothesizing that CAFs foster the niche by enriching the pro-tumor factors, an evidence-based study illustrated that an enhanced expression of TGFβ1 in a CAF-conditioned medium (CAF-CM) remained fundamental to the mesenchymal transformation of the non-CSC bladder cancer cells (Zhuang et al., 2015). Cancer cells induce pro-tumor CAF to establish a pro-favorable niche. TGFβ3 produced from head and neck squamous cell carcinoma (HNSCC) cells can stimulate CAFs to release POSTN, an ECM protein, augmenting the inclination of neoplastic cells towards altered plasticity and metastasizing (Qin et al., 2016; González-González and Periostin, 2018). CSC markers and stemness genes are elevated due to the binding of CAF-derived POSTN to protein tyrosine kinase 7 (PTK7) (Yu et al., 2018a). Likewise, prostate cancer cells turned externally administered bone marrow-derived mesenchymal stem cells (MSCs) into CAFs by TGFβ1 (Barcellos-de-Souza et al., 2016). Interestingly, these stem cell-derived CAFs can change the TME into an M2 (macrophage) phenotype dominant one by transforming residual monocytes. These CAFs can also release cardiotrophin-like cytokine factor (CLCF1) that primarily acts through the JAK-STAT pathway in a positive feedback loop to produce TGFβ and C-X-C motif ligand 6 (CXCL6) (Song et al., 2021). This positive feedback mechanism engages the CAFs to induce stemness by plentiful TGFβ in the TME (Song et al., 2021). In another example citing the role of CSCs in augmenting stemness factors in the TME, a similar paradigm comes into play to employ CAFs by CD24^+^CD49f^hi^ breast CSCs that trigger the production of components of ECM and pro-tumor paracrine factors in CAFs (Valenti et al., 2017). Valenti *et al.* (2017) (Valenti et al., 2017) demonstrated that Sonic hedgehog (Shh) derived from breast CSCs were responsible for activating CAFs that, in turn, supplemented the stemness (Valenti et al., 2017; Cazet et al., 2018). Recent research on the origins of chemotherapy resistance found IL-34 and macrophage colony-stimulating factor-1 (M-CSF-1) to be two of those (Baghdadi et al., 2018; Hama et al., 2020). IL-34, with the help of M-CSF-1, acted in a pro-survival way for the tumor cells by helping the monocytes grow alongside converting NFs to CAFs. These CAFs can transform these monocytes into immunosuppressive M2 macrophages, besides secreting stemness-promoting factors like Netrin-1 and FGF2 protein (Suh et al., 2016; Sung et al., 2019). The conversion into the M2 phenotype also inhibits the cytotoxicity of tumor antigen-specific CD8^+^ T-cells (Baghdadi et al., 2016; Giulianelli et al., 2019; Sung et al., 2019; Blondy et al., 2020). Other examples involve *in vitro* data, suggesting that the influence of HNSCC cells on the CAFs to produce Wnt3a increases cancer stemness (Le et al., 2019).

CAF-derived factors play critical roles in developing and maintaining CSCs by boosting stemness pathways. Hepatocyte growth factor (HGF), released by stromal cells, like fibroblasts, dramatically enhances stemness and EMT gene expression by increasing the expression of CD44^+^, CD47^+^, and CD90^+^ markers tissue (Yan et al., 2018a; Ding et al., 2018). A rise in the stemness markers and an increase in spheroid formation were demonstrated to be due to the activity of CAF-derived IL-6 through the STAT3 pathway (Xiong et al., 2018; Zhang et al., 2020). Other well-known stemness factors, like NANOG and ALDH, are similarly enhanced by the AKT pathway, which is controlled by the CAF secretome (Li et al., 2018). Furthermore, CAF is a crucial modulator of EMT in cancer cells due to the release of IL-6 (Raskov et al., 2021). A few other molecules causing EMT and stemness are Serglycin (SRGN), Annexin A1 (AnxA1), and Prostaglandin E2 (PGE2), all of which are derived from activated CAFs and act through paracrine signaling on the non-small cell lung cancer (NSCLC) cells, prostate cancer cells, and intestinal cancer cells respectively (Geary et al., 2014; Guo et al., 2017; Roulis et al., 2020). Moreover, exosomal miRNAs such as miR-378c, miR-143, and miR-21, derived from CAFs, play significant roles in EMT and the generation of stem-like cells (Donnarumma et al., 2017; Yang et al., 2017).

All these data allude to the critical role of the CAFs, being an intermediary, following commands from tumor cells to stimulate stemness. Nonetheless limited data in this domain necessitates further efforts to fill knowledge gaps.

### Role of CAFs in metastasis, migration, and invasion

The ability of CAFs to induce cancer invasion and metastasis is their most significant distinguishing attribute compared to NFs. Data suggests that CAFs remodel the ECM, leading to cancer progression through invasion and metastasis (Dumont et al., 2013b). Answering the controversy regarding whether the number of CAFs decreases or increases to cause the cancer cells to invade ECM, an exciting study employing a deep learning cell identification algorithm using TCGA suggested an increase in the number of CAFs in the TME (Shapcott et al., 2019). The CAF model differs from typically observed cancer cell metastasis in invadopodia-independent and MMP-independent matrix degradation processes (Cao et al., 2016; Glentis et al., 2017). The heterogeneity and plasticity of CAFs are also critical for invasion and metastasis. The interplay among the microbiota present in the TME portrays itself as the *cat’s-paw* for the metastatic cells. We have learned about two distinct CAFs, differentiated in terms of heterogeneity of surface markers (CD146^+/−^). CD146^-^ CAFs have been shown to enrich the TME with the production of proteins responsible for invasiveness, such as tenascin C (TNC), lysyl oxidase (LOX), and fibronectin 1 (FN1) (Brechbuhl et al., 2020). Earlier studies showed that CD146^+^ cells may represent a more mature pericyte subpopulation than CD146^-^ cells (Hassanpour et al., 2020; Manocha et al., 2022). CD146^-^ is a potent marker for risk analysis for lymph node metastasis, and patients with higher CD146^-^ cells have a poor prognosis (Brechbuhl et al., 2020). In convergence, the accumulated fibronectin (FN) in CAFs accelerates the invasion mainly *via* integrin-αvβ3. The assembled FN and the expression of αv and β3 integrins are directly proportional to the capacity to breach the basement membrane (BM) (Attieh et al., 2017). However, another integrin α_11_β_1_, a stromal cell-specific receptor for fibrillar collagens, indirectly aids in lung metastasis *via* facilitating trans-differentiation of CAFs (Navab et al., 2016).

It is broadly accepted that the tumor-promoting microenvironment in metastasis lesions emerges prior to the arrival of cancer cells, a process known as premetastatic niche (PMN) development (Fares et al., 2020). Soluble factors such as TGFβ, VEGF, and TNF, and EVs, like exosomes released by CAFs, promote PMN development. These factors then enter the circulatory system and spread to remote targets (Kong et al., 2019; Ping et al., 2021). Exosomes derived from CAFs contain non-coding RNAs (especially miRNAs) that regulate the cancer cells in a paracrine fashion (Yang et al., 2019; Li et al., 2021; Zhan et al., 2021). The differential distribution of these miRNAs in CAFs and NFs draws a thin line between their ability to manipulate the cancer cells to grow and invade distant tissues (Herrera et al., 2018). For example, miR-92a-3p expression was dramatically elevated in colorectal cancer (CRC) cells following phagocytosis of CAFs-derived exosomes (Hu et al., 2019). Again, a decrease in the miR-3188 levels in the CAFs-derived exosomes compared to that of NFs has been stated to have a role in turning on malignant phenotype in head and neck cancer cells (Wang et al., 2019).

POSTN^**+**^ CAFs have been demonstrated to enhance lymph node metastasis as well as boost cancer cell proliferation and invasiveness (Hong et al., 2010; Wei et al., 2021). They activated the integrin-FAK/Src-VE-cadherin signaling cascade to penetrate lymphatic endothelial barriers, culminating in metastatic dissemination (Hong et al., 2010). CAFs are also reported to facilitate metastasis depending upon the presence or absence of stanniocalcin-1 (STC1). Pena *et al.* (2013) (Pena et al., 2013) demonstrated that CRC cells failed to invade and migrate *in vitro* and *in vivo* when co-cultured with STC1-deficient CAFs and grown in an orthotopic mouse model, respectively (Pena et al., 2013).

The predominant stromal cells in the TME account for the primary ECM source and thus can easily be attributed to its novel ability to modulate the same. Glentis et al. (2017) demonstrated a crucial role of CAF-containing stroma in paving the matrix-metalloproteinases (MMP)- independent mechanism of invasion by the colon cancer cells (Glentis et al., 2017), in contrast with previous belief that MMP2 and -9 are pre-requisites for metastatic dissemination of cancer cells to distant locations (Taguchi et al., 2014). The activation of CAFs and expression of αSMA rest on the secretome of CAFs. According to Tang et al. (2016), the invasive tendency of breast cancer cells grown *in vitro* is caused by the downregulation of the miR-200s (miR-200 family), which is consistent with the prometastatic nature of CAFs. These studies also shed insight into the functions of miRNAs in the transdifferentiation of fibroblasts (Tang et al., 2016).

Although molecular aberrations prevail in cancer cells, however, epigenetic alterations and variable miRNA expression are mostly fundamental for the cancer-promoting properties of CAFs. Despite several early reports of p53 mutations in the stromal compartment, it is now widely believed that CAFs do not carry p53 mutations (Hu et al., 2005; Fukino et al., 2007; Bechtel et al., 2010; Zhao et al., 2012). However, an oncogenic *gain-of-function* is attained inside cancer cells, above and beyond ablating the tumor suppressive function, upon mutation in the *TP53* gene (Muller and Vousden, 2013). At the early stage of carcinogenesis, p53 functions as a *cell non-autonomous tumor suppressor* in the fibroblasts, in part by preventing the production and secretion of several molecules that might promote tumor growth (Kiaris et al., 2005; Moskovits et al., 2006; Addadi et al., 2010; Otomo et al., 2014). However, sustained cross-talk with the cancer cells induces trans-differentiation of their neighboring fibroblasts into CAFs (Kalluri, 2016). In agreement, Arandkar et al. (2018) have demonstrated the role of CAFs in invasion and metastasis correlating to an altered p53 function in the CAFs, suggesting that p53 acted as an essential modulator of the epigenetic machinery of CAFs in comparison with NFs. p53 was shown to have inherent, cell-autonomous, and unique characteristics imparted upon CAFs compared to NFs. A global transcriptome analysis using RNA sequencing data showed that 1,662 genes were expressed differentially in CAFs, supporting cancerous growth, proliferation, invasion, and migration. Cancer cells’ migratory characteristics were attenuated on silencing p53 in the CAFs (Arandkar et al., 2018).

### Role of CAFs in *angiogenesis*

New vasculature connectivity formation is essential because tumor cell proliferation and metastatic spread rely on optimal oxygen intake, supply of nutrients, and waste elimination (Lugano et al., 2020; Notohamiprodjo et al., 2021). The endogenous angiogenic factors responsible for the activation of neovasculature are namely VEGF, bFGF, TGFα, TGFβ, TNFα, angiogenin, PDGF, G-CSF, placental growth factor, IL-8, HGF, and epidermal growth factor (EGF) (Nishida et al., 2006). The role of CAFs in neovasculature to aid hypoxic tumor cells depends broadly on the positive and reciprocal feedback among CAFs and tumor cells. CAFs recruit endothelial cells and stimulate vascularization, triggering angiogenesis for the nutrient supply to cancer cells (Sewell-Loftin et al., 2017). The connective tissue growth factor (CTGF) expression is essential for boosting microvessel density and attracting endothelial cells in experimental xenograft models of CAF-mediated angiogenesis through the expression of SDF-1/CXCL12 (Denton et al., 2018). Apart from that, there is another indirect role of CAFs in triggering angiogenesis. As discussed, CAFs regulate and modify the ECM in favor of the cancer cells to grow and metastasize. Such modifications include the upregulation of MMP9 and MMP13, leading to the secretion of pro-angiogenic growth factors like VEGF (Boire et al., 2005; Vosseler et al., 2009).

Overexpression of SDF-1/CXCL12 by αSMA^**+**^ CAFs and simultaneous downregulation of mDia2 protein triggered pro-angiogenic secretome profile in breast cancer (Orimo et al., 2005; Yu et al., 2014; Dvorak et al., 2018). Apart from secreting VEGF, CAFs recruit endothelial progenitor cells (EPCs) in the breast cancer TME resulting in angiogenesis (Orimo et al., 2005; De Francesco et al., 2013). In line, Wang et al. (2014a) showed malignancy and angiogenesis in nasopharyngeal carcinoma (NPC) in a VEGF/CXCL12-CXCR4 dependent manner, and the presence of EPCs was also confirmed with CD133^**+**^ cells in the tumor stroma. Two independent research groups reported that the SDF-1/CXCR4 cascade was also employed by αSMA^+^ CAFs following HIF-1α mediated conversion into CAFs from NFs (Zagzag et al., 2005; Errarte et al., 2020). Recent research unveiled an exciting correlation of αSMA and CD90 of CAFs with neighboring HCC tissue expression of placental growth factor (PGF) (Liu et al., 2020a). PGF causes blunted anti-tumor immunity by M2 polarization and creates a pro-angiogenic environment by triggering the inflammatory markers, NF-κB and COX2 in CAFs (Albonici et al., 2019). With the help of TCGA analysis, Liu et al. (2020a) showed that the co-expression of PGF and CD90 in the tumor milieu could be directly correlated to the angiogenesis markers CD31, CD34, and CD105 leading to poor prognosis of HCC patients.

In a study by Erez et al., 2010, PDGFRα^**+**^ CAFs were sorted from a squamous cell carcinoma tumor milieu (SCC) to decipher the role of inflammatory secretome and others in triggering pro-angiogenic signal transduction. Two different pathways could be decoded to reach VEGF expression: directly through OPN and CYR61 expression or secondarily *via* the expression of inflammatory genes CXCL1/2/5 (Erez et al., 2010).

CAF-secreted exosomes containing miRNAs play a significant part in angiogenesis too. miR-21 was widely accepted to induce VEGF-mediated pro-angiogenic signaling in an autocrine fashion (Asangani et al., 2008; Wu et al., 2017). Nevertheless, the CAF-exosomal miR-21 might also add to the cumulative induction in a paracrine style (Yang et al., 2017). Ten-eleven translocation 2 (TET2) was found to be the target of cancer cell-secreted miR-210 in CAFs, which has been implicated in the pro-angiogenic switch. A recent analysis revealed that miR-210 had the capacity to elevate the expression of some pro-angiogenic factors, including MMP9, FGF2, and VEGFa, in CAFs, by activating the JAK2/STAT3 signaling pathway (Fan et al., 2020). Furthermore, miR-135b-5p upregulation in the CAF-derived exosomes supported the CRC cell migration and angiogenesis (Yin et al., 2021).

### Role of CAFs in extracellular matrix production and remodeling

The English surgeon Stephen Paget (1855-1926) postulated the “seed and soil” theory a century ago to describe the role of tumor stroma in the growth and dissemination of cancerous cells. The “seed” is the cancer cell, while the stroma or the microenvironment is considered the “soil” for the “seed” (Paget, 1889; Ribatti et al., 2006). Current knowledge suggests that the soil requires positive reciprocal feedback from the seed or that it sometimes needs to be educated about the needs of the seed. For example, we may consider one of the many different means discussed that resulted in a transdifferentiation mesenchymal to mesenchymal transition (MMT) of NFs to CAFs (Sahai et al., 2020a; Ping et al., 2021). p53 non-autonomous expression pattern in the CAFs is believed to be due to the education imparted by the cancer cells. Furthermore, qRT-PCR further confirmed that MMP1, MMP3, and MMP10 mRNA were upregulated in CAFs in a largely p53-dependent manner, precisely opposite to NFs (Arandkar et al., 2018).

CAFs are considered the key ECM modulator in the tumor stroma, influencing tumor development, intravasation, migration, extravasation, and metastasis (Sahai et al., 2020a). Many malignancies are characterized by increased collagen synthesis (Liu et al., 2019a). Increased collagen synthesis and cross-linking, in particular, are linked to increased tumor stiffness and advancement (Xu et al., 2019). A cancer spheroid formation model of ovarian cancer confirmed the role of CAFs in influencing collagen type I, particularly by expressing versican, which promoted cancer invasiveness *via* the TGFβ pathway (Yeung et al., 2013). In addition, CAF-derived laminin was shown to interact with integrin α_6_β_4_ to trigger the migration of cervical cancer cells (Fullar et al., 2015). Following conversion into CAFs, contractile capabilities increase as αSMA and vimentin expression levels rise. That gives rise to its morphological modification by giving it a stellate shape (Kalluri, 2016), and several different soluble and insoluble secretions, including ECM proteins, are observed to be significantly intensified. Fibronectin is deposited first by the CAFs, which produces and intensifies intracellular tensions *via* increased interaction with actin filaments. In the case of wound healing, a positive feedback loop keeps the fibroblasts activated, and the translocation of yes-associated protein (YAP) into the nucleus triggers overexpression of αSMA (Kollmannsberger et al., 2018). Before producing and conducting tensile strength to the matrix to remodel it, the contractility of the CAFs needs to get amplified. That occurs through the activation of the Rho-ROCK-Myosin II signaling cascade by the signals from newly synthesized ECM and subsequent amalgamation of αSMA with actin-myosin fibers (Gaggioli et al., 2007; Provenzano and Keely, 2011). The CAFs modify the composition by modulating the amount and expression of MMPs, and mechanically, they change the physical properties of the ECM by modifying its organization and stiffness (Jang and Beningo, 2019).

Participation of additional fibroblasts generates a negative feedback loop to reduce the fibronectin/collagen I ratio leading to the relaxation of fibronectin fibers and CAFs entering quiescence. In contrast to the physiological context, fibronectin fibers are not let relaxed to resume quiescence; instead, the fibronectin zones are altered as a preventive measure against relaxation, which, in turn, leads to continuous ECM remodeling in the tumor context (Cox and Erler, 2011; Pickup et al., 2014; Leight et al., 2017; Barbazán and Matic Vignjevic, 2019). These secretions allow the CAFs to stay activated and communicate with the neighboring cells, such as endothelial cells and immune cells, in the stroma. Several trials targeting the alliance between CAFs and ECM modifications in recent years have unraveled that it not only supports the cancer progression, but the efficiency of targeting also depends on the tissue and carcinogenesis stage-specific context (Amakye et al., 2013; Van Cutsem et al., 2020). In some cases (e.g., breast, colon, pancreatic, stomach cancer), the stromal fraction of solid tumors can vary widely, amounting to 60%–90% of the overall tumor mass (Ronnov-Jessen et al., 1996; Dvorak, 1986; Powell et al., 2005; D'Arcangelo et al., 2020). In fact, TME can be very much influenced by the CAFs through the formation of severe desmoplastic reactions creating a desmoplastic TME (Liu et al., 2019a; Zeltz et al., 2020b). Cancer dissemination from PDAC to other organs can be attributed to desmoplasia (Shimosato et al., 1980; Halvorsen and Seim, 1989a; Halvorsen and Seim, 1989b; Hasebe et al., 2002; Iacobuzio-Donahue et al., 2002; Koliopanos et al., 2002; Caporale et al., 2005). However, in the case of CRC, the role of desmoplasia is not as straightforward as PDAC (Hewitt et al., 1993).

In a previous study by Erkan et al., 2008, increased stromal activity (but not stromal density) and diminished collagen deposition were correlated with a worse prognosis. In contrast, lower stromal activity (indicated by low αSMA expression) and increased collagen deposition were synchronized with a better prognosis (Erkan et al., 2008). This came with a contradiction against the dogma that collagen-induced signals highly favor the aggressiveness of cancerous growth (Whatcott et al., 2015; Insua-Rodríguez and Oskarsson, 2016). In another study, Bever *et al.* (2015) (Bever et al., 2015) illustrated the correlation between stroma density and patient survival and stroma activity using a computer-aided quantitative method in a cohort of 66 PDAC patients under adjuvant medication following a pancreaticoduodenectomy. In line with this, Chen et al. (2021) demonstrated that deletion of type 1 collagen in αSMA^+^ myofibroblasts exaggerated PDAC, probably due to high stromal activity. More studies in the field of collagen arrangements in the stroma found that rather than the collagen quantity (presence or absence of collagen), it is the collagen quality (thickness and alignment) that is indicative of poor disease prognosis (Drifka et al., 2016; Laklai et al., 2016). That concludes that activation of CAFs and ECM modification is equally essential for cancer progression and metastasis.

The ECM stiffness gives rise to invadopodia formation from actomyosin beams in cancer cells. The rate of invadopodia formation and matrix degradation depends upon the contractility of actomyosin beams (Aung et al., 2014; Jerrell and Parekh, 2014). The invadopodia are enriched with MMPs that form passages for the cancer cells to infiltrate into the ECM by degrading the same (Kessenbrock et al., 2010; Schoumacher et al., 2010). However, recent research reported that CAFs could degrade the ECM in a manner independent of invadopodia. It utilizes the tubular organization, employing MT1-MMP (MMP-14) or MMP-2 protein to remodel the matrix in favor of tumor cell invasion and metastasis (Cao et al., 2016). SPARC, secreted from CAFs, supports the pro-tumor mechanisms in many cancer types. Through the differential nature of executions in a few cancer types, it aids cell invasion by enhancing MMP expression by CAFs as well as by monocytes (Tremble et al., 1993; Neuzillet et al., 2013).

CAFs, educated *via* TNFα and NF-κB pathway by the p53 mutant cancer cells, in the stroma of metastases have been shown to release significantly high quantities of perlecan in order to attract cancer cells (Pereira et al., 2019). Thus, the cancer cells, CAFs, and immune cells secrete large amounts of VEGF, PDGF, and FGF-β which add to the basement membrane’s (BM) pre-stored growth factors and are finally released after degradation by the MMPS (Kalluri, 2003). The disorders created in the BM enhance tumor cell invasion by helping pro-angiogenic mechanisms.

Moreover, CAFs may indirectly influence inflammatory cell recruitment as well as functioning *via* ECM deposition and matrix remodeling (Rubin et al., 1995). CAF-directed ECM alteration is important for monocyte and other myeloid cell recruitment. The stiffness of the ECM supports the release of chemoattractants like CCL2 and colony-stimulating factor-1 (CSF-1) from CAFs and tumor cells (Acerbi et al., 2015; Nielsen et al., 2016). The composition, density, and organization of ECM support the passage of immune cells through the stroma. While hyaluronan and fibronectin prevailing stroma can promote T-cell migration through ECM, versican and tenascin can inhibit the same (Loike et al., 2001; Evanko et al., 2012; O'Connor et al., 2012; Bollyky et al., 2011; He and Baum, 2004). On the other hand, the pro-tumorous M2 macrophages can be recruited and blocked by collagen and hyaluronan-rich and SPARC-rich matrices, respectively (Kuang et al., 2007; Kobayashi et al., 2010; Sorokin, 2010; Iijima et al., 2011).

### Role of CAFs on immune cells

**FIGURE 3:**
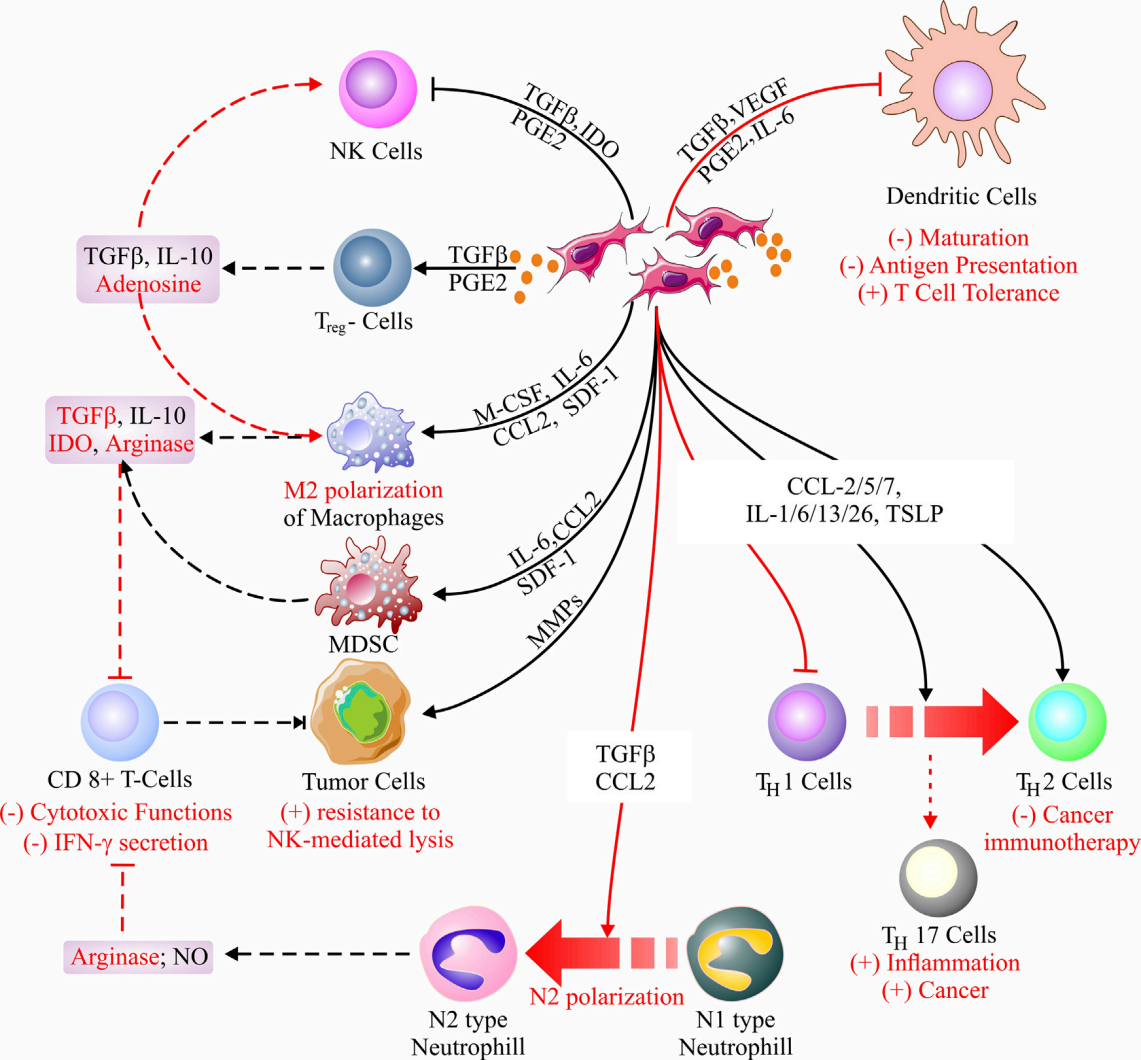
Cancer-associated fibroblast (CAF) immunomodulatory mechanisms. CAFs-derived secretome primarily alters the immune microenvironment around the stroma by infusing immunosuppression. Inhibition of NK cells, generation of T_reg_ cells, and M2 polarization of macrophages are the key reasons behind the immunosuppression. Besides, CAFs can block antigen presentation by inhibiting DCs, largely impeding cell-mediated immunity. In addition, CAF-secretome is responsible for N1 to N2 conversion, thereby minimizing the T-cell (CD8^+^)-mediated cytotoxicity against tumor cells. Moreover, CAFs are known to regress the impact of cancer immunotherapy and favor tumor progression *via* the reduction of T_H_1 cells through their conversion into T_H_2 cells and generation of T_H_17 cells, respectively (Dikov et al., 2005; Kitamura et al., 2005; Flavell et al., 2010; Allard et al., 2016; Hashimoto et al., 2016; Kitamura et al., 2017; Mantovani et al., 2017; Takahashi et al., 2017; Cheng et al., 2018; Nakamura et al., 2018; Qian et al., 2018; Liu et al., 2019a; Monteran and Erez, 2019a; Masucci et al., 2019; Owusu-Ansah et al., 2019; Zhang et al., 2019; An et al., 2020; Veglia et al., 2021).

CAFs and immune cells are the most prevailing cell types within the tumor stroma. Among the immune cells, CAF-derived secretome affects many leukocytes, including CD8^+^ T-cells, T_reg_ cells, and the most abundantly present macrophages (Figure 3). Considering their vast role in TME in tumor progression, invasion, metastasis, and acquired resistance to therapy, the macrophages (henceforth will be designated as ‘tumor-associated macrophages’ or TAMs) will cover a big part of our discussion.

Besides TAMs, CAFs have a multifaceted level of interactions with natural killer (NK) cells, dendritic cells (DC), T lymphocytes, myeloid-derived suppressor cells (MDSCs), and tumor-associated neutrophils (TANs), forming a complex tumor-immune interface (An et al., 2020). As discussed in previous sections, the putative impact of the CAF-derived secretome affects immunosurveillance negatively. Exceptions include the interaction of DC cells from hepatocellular carcinoma (HCC), bone marrow cells, and CAFs in a co-culture system generating IL-1β, IL-6, and IL-12p70, which subsequently activates cytotoxic T lymphocytes (T_C_ cells) to release interferon (IFN-) α, and IFN-γ, eliciting an anti-tumor effect (Qian et al., 2018). The interactions with other cells have been discussed in-depth in previous sections detailing the functions and diversity of CAFs (Fearon, 2014; Lakins et al., 2018a; Costa et al., 2018; Elyada et al., 2019). Zhang et al. (2019) have shown that CRC-derived CAFs could upregulate the expression of VCAM1 and secretion of IL-8, thereby recruiting and polarizing macrophages into the M2 phenotype, culminating in diminished NK cell function. Synergism between CAFs and TAMs in crafting an immunosuppressed TME was shown to regulate tumor-infiltrated NK cells (Zhang et al., 2019). Next to NK cells, Cheng et al. (2018) reported a novel mechanism regarding the effect of CAFs on tumor-associated neutrophils (TANs). IL-6 secreted from the CAFs induces STAT3/PD-L1 signaling pathway in TANs triggering chemotaxis and anti-apoptosis mechanism triggering an increase in the number of neutrophils in the TME. A previous report indicated that an N1/N2 phenotypic transition is attributable in the TANs, where N1 is considered to have anti-tumor activity, and N2 is regarded to be associated with immunosuppression, and pro-tumor cellular activities, by DNA instability or by secretion of chemokines and cytokines (Masucci et al., 2019; Lau et al., 2022). The prevalence of the N2 phenotype correlates with poor patient prognosis (Masucci et al., 2019).

Some clues indicate a clear association of CAFs with the polarization of TAMs. CAF-derived secretome includes a plethora of soluble effector molecules, and current studies indicate that CAFs are the regulators of different cell-mediated immune responses (Monteran and Erez, 2019b). In agreement with this, CAFs were able to recruit and differentiate macrophages into TAMs (Ziani et al., 2018; Hegab et al., 2019). CAFs are involved in MCP-1-mediated macrophage infiltration in skin tumors, nourishing chronic inflammation (Zhang et al., 2011). Likewise, podoplanin^+^ CAFs could recruit and differentiate monocyte into CD204^+^ TAMs (Sakai et al., 2018). With the key monocyte chemotactic cytokines MCP-1 and SDF-1, the CAFs from invasive human breast cancer were able to transform monocytes into M2-type pro-tumoral macrophages, changing the myeloid lineage structurally as well as functionally (Gok Yavuz et al., 2019). Interestingly, the newly transformed M2-type macrophages (TAMs) can activate CAFs in a positive feedback loop and promote cancer progression (Ueshima et al., 2019). The ephemeral reciprocation in the action of CAFs and TAMs was justified by Hashimoto *et al.* (2016), where the group demonstrated the advent of CAFs from bone marrow-derived MSCs, their role in increased invasiveness of TAMs, finally culminating in the generation and invasion of more CAFs (Hashimoto et al., 2016; Zheng et al., 2017). In addition to SDF-1, CAFs secrete CXCL14 in prostate cancer, which accelerates the recruitment of monocytes and further leads to their polarization into M2 TAMs (Augsten et al., 2014; Comito et al., 2014). Another mechanism of M2-phenotypic conversion was demonstrated in CRC, *in vitro*, where CAFs-derived IL-6 and IL-8 were found responsible for the conversion (Kim et al., 2012). ER^-^/PR^-^/HER2^-^ triple-negative breast cancer stroma-derived CAFs secrete CXCL16 that attracts monocytes and aids their stromal activation (Allaoui et al., 2016). Adenosine secretion by CAFs warrants great potential for monocyte differentiation and polarization into the M2 phenotype (Regateiro et al., 2012; Della Latta et al., 2013; Allard et al., 2016; de Leve et al., 2019). To describe the TME state that supports the recruitment and conversion of macrophages, Nakamura *et al.* (2018) deciphered that a hypoxic environment, substantiated by the marker carbonic anhydrase IX, contains both podoplanin^+^ CAFs as well as CD204^+^ TAMs (Nakamura et al., 2018). CD204^+^ TAMs are associated with immune suppression. Evidence-based data support the involvement of FAP^+^ cells in activating inflammatory STAT3 cascade *via* the uPAR-FAK-*c*-Src-JAK2 pathway. Myeloid-derived suppressor cells (MDSCs) are recruited to the tumor stroma to promote immunosuppression. CCL2, derived from FAP^+^ CAFs, is considered responsible for the CCR2-dependent immunosuppression by the MDSCs (Yang et al., 2016a; Kumar et al., 2017). CD163^+^ TAMs infiltrated the tumor stroma in triple-negative breast cancer and nasopharyngeal cancers, indicating a close association between CAFs and TAMs (Yu et al., 2018b; Zhou et al., 2018). In addition to direct mechanisms involved in M2 polarization by CAFs, there lies an indirect path as well. The pancreatic stellate cells (PSCs), considered the critical source cells of CAFs in PDAC, could stimulate IL-13-secreting mast-cells, promoting M2 macrophage polarization (Ma et al., 2013; Varricchi et al., 2017).

The crosstalk between CAFs and TAMs (M2-type), along with other cells, and their positive and reciprocating aid towards pro-tumorous, immunosuppressed TME greatly facilitated tumor growth and immune evasion (Hashimoto et al., 2016; Gunaydin, 2021). Further, the CAF-TAM communication was also helpful in advancing cancer with angiogenesis and metastasis (Comito et al., 2014; Doak et al., 2018). CAF/TAM milieu is associated with the generation of perlecan-rich desmoplastic stroma at metastatic sites (Brasil Da Costa et al., 2020). To aid metastasis, CAFs recruit EPCs (Orimo et al., 2005; De Francesco et al., 2013; Wang et al., 2014a) that aid in bringing in epigenetic modifications in the cancer cells in collaboration with the CAFs. The modification leads to a *mesenchymal-to-amoeboid transition* (MAT), helping in the metastatic spread of tumor cells (Giannoni et al., 2013). Linde *et al.* (2018) illustrated the role of CAF-educated TAMs (CD206^+^) in metastasis. The CD206^+^ M2 macrophages were able to downregulate the E-cadherin in breast cancer cells, promoting EMT (Linde et al., 2018). Moreover, CAFs-secreted IL-33 triggered the M2 TAMs to upregulate the MMP-9 expression, enabling ECM modification (Andersson et al., 2018). There is substantiating evidence indicating the ability of CAFs to induce EMT, invasion, and metastasis (Karagiannis et al., 2012).

Stepping into the roles of CAFs in immune evasion, the primary conception leads us to infer that the M2 phenotypic milieu in the TME is key to immune evasion for the tumor cells. Moreover, CAF-derived secretome includes TGFβ, IL-10, and arginase I to add to the immunosuppressive profile (Takahashi et al., 2017). By secreting adenosine, an immunosuppressive metabolite, the CAFs enhance immunosuppression and myeloid differentiation, proliferation, and invasion (Leone and Emens, 2018). Moreover, the synergism of CAFs and TAMs can hinder adaptive immunity by interrupting antigen presentation. Besides TGFβ and IL-6, CAF-secretome includes tryptophan 2,3-dioxygenase (TDO2), indoleamine-2,3-dioxygenase (IDO), and VEGF that can block DC maturation, a critical antigen-presenting cell (APC). The absence of proper antigen presentation calls for T-cell anergy, a crucial event disrupting the cell-mediated immune response (Gabrilovich et al., 1996; Dikov et al., 2005; Kitamura et al., 2005; Flavell et al., 2010; Kitamura et al., 2017; Ziani et al., 2018; Liu et al., 2019a).

Therefore, the orchestration between the primary stromal cells in association with CAFs promotes the tumor cell *via* immune evasion and immunosuppression to boost the pro-tumorous TME for enhanced proliferation, growth, invasion, and metastasis of distant tissues, which ultimately leads to disease progression.

### Role of CAFs in cancer cell metabolism

Metabolism and metabolic crosstalk are indispensable parts of cancer. The secreted amino acids and other metabolites build a dialogue between the tumor cells and the stromal cells (Kay and Zanivan, 2021). Little is known about these interactions, and it remains open to answering how the CAF metabolism should be targeted for better patient prognosis.

Previous data suggest that CAFs show a characteristic hyper rate of glycolysis, and a proportional rate of autophagy is also typical. This event results in the release of a large amount of lactate, amino acids, and ketone bodies, which are subsequently taken up by tumor cells, helping them grow at a higher rate. The symbiotic dialogue between CAFs and tumor cells is the initial evidence of metabolic crosstalk (Pavlides et al., 2009; Whitaker-Menezes et al., 2011; Guido et al., 2012; Sousa et al., 2016). Aside from lactate, recent reports indicate pyruvate to be the major contribution of CAFs to the metabolome of the stroma as well. Significantly high secretion from breast CAFs and PDAC-CAF conditioned media substantiated the claim for a pyruvate-rich metabolome (Becker et al., 2020; Kerk et al., 2020). Kerk et al. (2020) described that the CAF-secreted extracellular pyruvate maintained redox homeostasis and was beneficial in developing resistance against mitochondrial inhibitors in pancreatic cancer. Strategic aid from pyruvate was again validated by the reports of lymphoma cell survival and ECM remodeling by metastatic breast cancer cells (Sakamoto et al., 2019).

In the case of pancreatic cancer, the quiescent stellate cells get converted into activated fibroblasts (CAFs) and release the stored lipid to assist as a signaling molecule for biomass production. For example, lysophosphatidic acid released from CAFs activates the PI3K/Akt signaling cascade in favor of cancer cell proliferation (Auciello et al., 2019).

The activity of cancer cells-derived-TGFβ and oxidative stress in rewiring CAF metabolism, inspiring glycolysis, and autophagy is widely accepted (Martinez-Outschoorn et al., 2010; Guido et al., 2012). The TGFβ assists the branched-chain keto acid production in CAFs. Branched-chain keto acids are used by cancer cells as carbon and nitrogen sources and were also deciphered from the overexpression of branched-chain amino-acid aminotransferase (BCAT) 1 (Zhu et al., 2020). Autophagy-derived alanine from CAFs is, reportedly, taken up by the PDAC cells to aid the TCA cycle, where the initial starting molecule is derived from pyruvate (Sousa et al., 2016; Auciello et al., 2019; Sanford-Crane et al., 2019). So metabolic convergence between the different sources can be met to justify the proliferation and growth of cancer cells.

Breast cancer-derived exosomes could activate the proto-oncogene *MYC* in the stromal fibroblasts in a positive and reciprocal feedback loop, as the activated CAFs then secret exosomal factors to be taken up by the cancer cells. This results in cancer cell proliferation *via* an increase in glucose and glutathione metabolism (Zhao et al., 2016; Yan et al., 2018b). Other metabolic exchanges include glutamine-glutamate exchange that benefits both CAFs and cancer cells. Inhibition in the synthesis of glutamate or glutamine showed reduced tumor growth (Yang et al., 2016c). As mentioned before, if not starvation, nutrient deficiency is a common condition for cancer cells. CAFs have been reported to support prostate cancer cells under glutamine deficiency in an ATF4-dependent manner *via* pyruvate carboxylase-asparagine synthase overexpression (Linares et al., 2017), where stromal asparagine was used as an alternative source of nitrogen. In stiff ECM, CAFs enhance their rate of glycolysis and oxidative phosphorylation. At this stage, CAF-derived aspartate is taken up by cancer cells, and in convergence, cancer cells-derived glutamate is taken up by the CAFs. Interestingly, this feedback loop stimulating cancer cell growth and invasion offers a common target glutaminase-1 (GLS1) to neutralize both pathways (Bertero et al., 2019).

For CAFs, creating a hypoxic environment is key to nurturing and expediting the growth of cancer cells with more glycolytic enzymes in the stroma. In addition, CAF autophagy also nurtures the metabolic needs of the proliferating cells by delivering lactate and other metabolites (Martinez-Outschoorn et al., 2010; Zhang et al., 2015). An entire epigenetic shift is responsible for adopting the pro-tumorigenic qualities of the TME. It is believed that hypoxia leads to a pro-tumorous gain of characters that include changes in gene expression patterns of CAF markers and ECM components *via* NNMT (Nicotinamide N-methyltransferase) mediated hypomethylation. Such changes were reported in ovarian CAFs (Eckert et al., 2019). Breast CAFs also supported a similar paradigm by acquiring hypomethylation of promoters of genes responsible for glycolytic enzyme synthesis (Becker et al., 2020). An upregulated NNMT in ovarian, colorectal, and gastric cancers indicates an active role of the enzymes in hypoxia-related hypomethylation (Eckert et al., 2019; Song et al., 2020; Zhang et al., 2021). These suggest that an epigenetic shift occurred, altering the CAFs to be pro-tumorous and remain active even in the absence of the primary inducer.

The function that gets affected by the altered metabolism of CAFs is the immune response against the tumor cells. Netrin G1 (NTNG1) is known for its role in glutamate and glutamine synthesis by CAFs, which is further taken up by cancer cells to expedite their energy needs. NTNG1 is also associated with immunosuppressive cytokine synthesis by CAFs (Kay and Zanivan, 2021). Therefore, the metabolism of CAFs has a multifaceted role inside the stroma, including nutrient supply to cancer cells and pro-tumorigenic immunomodulation.

## Role of CAFs in therapy resistance

Studies investigating various types of CAF-induced resistance to chemotherapy, radiotherapy, immunotherapy, and hormone therapy have revealed promising avenues for targeting CAFs and their effect on neighboring cells. CAF-induced resistance includes molecular mechanistic cyclization stimulation in nearby cancer cells, amplification of tumor-promoting secretome, evasion of immune checkpoint regulation, and enhanced paracrine/autocrine signaling or feedback loops that either directly or indirectly affect total tumor resistance. These events lead to tumor development and treatment resistance (Erdogan and Webb, 2017; Hilmi et al., 2020; Linares et al., 2020; Wessolly et al., 2022). Certain populations of CAFs are presumed to mediate the formation of an environment that is tumor-promoting and therapy-resistant (Simon and Salhia, 2022). Given that CAFs are the predominant component of the TME and exert a significant influence on cancer cell sensitivity to anti-cancer therapies, understanding the mechanisms of inhibition and resistance should be a prioritized area of research for sustaining therapy efficacy and overall patient survival.

### CAF-induced chemotherapy resistance

The main categories of overall chemoresistance induced by CAFs include ECM remodeling, paracrine signaling, induction of stem-like properties in cancer cells, metabolic manipulation, modulation of the immune environment in the TME, and exosomal shuttling between tumor cells and CAFs.

CAF-induced chemotherapy resistance involves multifaceted processes that generate a physical barrier by modifying the extracellular matrix (ECM). This barrier reduces drug accessibility to tumor cells, activates pro-survival signaling pathways, and inhibits apoptotic signaling pathways. ECM remodeling by CAFs may also result in increased EMT and stem-like properties, aggressive cancer cell transitions, epigenetic modulation, and general modulation of the crosstalk between breast tumor cells and stromal components in the TME (Maji et al., 2018). CAFs secrete type 1 collagen, which inhibits chemotherapeutic drug absorption in solid tumors (Luo et al., 2015).

Studies *in vitro* indicate that stromal-derived paracrine signaling can increase cancer cell survival following chemotherapy (Valkenburg et al., 2018). Chemotherapy-induced DNA damage in CAFs has been observed to increase the expression of various inflammatory, angiogenic, or EMT-inducing signals (HGF) (Vickman et al., 2020). There are cases of chemotherapeutic drugs activating NFs and promoting tumorigenic CAFs that are involved in paracrine signaling. For example, Hedgehog-GLI signaling induces stemness in cancer cells which can result in chemoresistance (Peiris-Pagès et al., 2015). CAF-mediated TGFβ signaling, along with other pro-EMT and tumor-promoting signaling pathways, causes breast tumor cells to resist chemotherapy and become more aggressive. Studies identifying pro-tumorigenic CAF subpopulations have contributed to expanding our scope of identifying paracrine signaling molecules and pathways. Han *et al.* (2021) investigated the molecular mechanism of paclitaxel resistance, a chemotherapeutic drug, in TNBC and correlated chemotherapy resistance in TNBC PDX models to JAK2 signaling and an enriched cCAF subpopulation (Han et al., 2021a).

The induction of stemness in cancer cells has been a crucial aspect of therapy resistance in all types of cancer. After treatment, the risk of repopulation and relapse is due to the ability of stem-like cancer cells to resist anti-cancer therapies. The evidence that CAFs are also a cause of inducing CSC populations in tumors is essential for comprehending the methodologies of chemotherapy resistance and preventing relapse. Su *et al.* (2018) demonstrated that the cell membrane proteins CD10⁺ and GPR77⁺ are responsible for the maintenance of stemness in breast CSC populations (Su et al., 2018). These populations are positively correlated with the expression of CD10⁺ GPR77⁺ CAFs, revealing that this subset of CAFs maintains stemness through TME modulation (Su et al., 2018).

Yu et al. (2017) described a stromal GPER-mediated drug resistance and increased mitochondrial activity in the reprogramming of breast cancer energy metabolism. The stromal environment is particularly acidic, but the relationship between lower pH and CAF-secreted lactate can also be associated with chemoresistance through metabolic reprogramming (Tavares-Valente et al., 2013). In agreement with this, the upregulation of Ras signaling in CAFs correlates with an increase in glutamine synthesis, which supports the mitochondrial metabolism of cancer cells and renders them more resistant to chemotherapy, particularly drugs that inhibit androgen signaling (Mishra et al., 2018). Understanding the mechanisms by which CAF influences the metabolism of tumor cells is a promising strategy for addressing issues of therapy resistance and cancer severity.

NFs play an integral role in immune response, and CAFs exploit this function to facilitate cancer cell immune evasion. NK cell abrogation is achieved by exposure to CAF-derived TGFβ through miR-183 mediated DAP12 transcription interruption, thus resisting chemotherapy and aiding cancer cell survival (Powell and Huttenlocher, 2016). The secretion of soluble factors has extensive effects on the EMT, proliferation, migration, and stem-like properties of cancer cells, as well as a prominent influence on immune response and evasion. CXCL secreted by CAFs recruits TAMs, stimulating cancer progression and chemoresistance (Shien et al., 2017). Due to its immunomodulating effects, the clinicopathological significance of CAF PD-L1 expression in TNBC has been identified as a reliable predictor of treatment response and prognosis (Yoshikawa et al., 2021). Interferons, which play a crucial role in immunomodulatory functions, are modulated by CAFs resulting in therapy resistance and tumor progression. Activation of IFN signaling induced by CAFs in claudin-low TNBCs resulted in chemotherapy resistance, as demonstrated by Broad *et al.* (2021) (Broad et al., 2021). Targeting IFN receptors and other signaling axes involved in CAF-dependent chemoprotection *via* immunomodulatory functions makes it possible to find more efficacious means of enhancing treatment outcomes. CAF-derived chemokines such as IL-8 or CXCL8 recruit immunosuppressive cells to the TME, consequently enabling effective tumor progression, angiogenesis, and EMT (Han et al., 2021b). Promoting immunosuppression by CAFs in the TME can result in resistance to chemotherapy and other therapies (Hu et al., 2021). CAFs also affect the ratio of immune cells within the TME, notably by increasing the proportion of FOXP3⁺ T-cell T_reg_ to CD8⁺ T lymphocytes, which leads to poor treatment outcomes and a diminished antitumor immune response (Mhaidly and Mechta-Grigoriou, 2021). The mechanisms of CAF immunomodulatory pathways may be crucial to our comprehension of CAF-influenced chemotherapy resistance.

Cancer cells have been seen to have gemcitabine and paclitaxel resistance in pancreatic cancer due to an association between CAFs, TAMs, and stromal-derived insulin-like growth factors (Ireland et al., 2016). Conversely, breast cancer cells increased chemotherapy efficacy by blocking the insulin-like growth factors from stromal components (Ireland et al., 2018). A possible counter to this is to target GPR77 membrane proteins in CAFs to promote chemosensitivity and reduce CSC count (Su et al., 2018). It has also been found that *in vitro* VCAM-1 knockdown in breast cancer cells reduces the proliferation and migration of IL-6-influenced breast cancer cells, thus increasing chemosensitivity (Wang et al., 2014b). Other methods of countering chemoresistance, such as aspirin treatment, have been reported to be effective as well, although their relationship to CAF-induced stemness still needs to be explored (Saha et al., 2016). More focus needs to be paid to how autophagy influences TME. To combat the chemoresistance brought on by CAFs, small compounds that target the autophagy-related core machinery may be a viable option. In a PDAC animal model, CAF depletion *via* disruption of the Hh signaling or injection of hyaluronan improved chemotherapy (gemcitabine) delivery (Olive et al., 2009). CAF-targeting strategies have shown progress in our understanding of how to increase chemotherapy efficacy and decrease overall tumor progression.

### CAF-induced hormonal therapy resistance

It has been well established that hormone imbalance, specifically endogenous estrogen and progesterone, can cause breast cancer *via* receptor-dependent and -independent mechanisms (Trabert et al., 2020). In addition to other secreting growth factors, cytokines, and proteases, estrogen can be secreted from CAFs and may be used to predict the efficacy of endocrine therapy and treatment response (Yu et al., 2017; Ruocco et al., 2018). Adjuvant endocrine therapy is an effective strategy against estrogen receptor-positive and other related types of breast cancer, such as luminal A, luminal B, or HER2⁺ (Schiavon and Smith, 2014; Burstein et al., 2019). In luminal breast cancer, microvesicle-mediated miRNA transfer transforms non-CSCs into CSCs that induce resistance to most therapies, including hormonal therapy. An example of this involves a study confirming the role of CAFs in the presence of hormone therapy resistance by modulating hormonal receptors or activating signaling pathways such as PI3K/AKT and MAPK/ERL1 and ERL2 signaling axis involved in tamoxifen resistance (Martinez-Outschoorn et al., 2011). Anti-estrogen resistance in breast cancer was found to be overcome by inhibiting mitochondrial function in breast cancer cells (Martinez-Outschoorn et al., 2011). Stromal factors influence the TME, and CAF-induced β1 integrin signaling promotes tamoxifen resistance in breast cancer (Pontiggia et al., 2012). Exosomal or vesicular transfer between tumor cells and CAF secretome frequently results in an active feed-forward loop involving CAFs and tumor cells that increases tumor-promoting CAF activation and aggressive features in cancer cells. Specifically, Cosentino et al. (2020) discovered that miR-9-mediated inhibition of EFEMP1 contributed to the upregulation of pro-tumor CAF phenotypes.

Developing methods to prevent the formation of CAF-influenced therapy-resistant tumor cells can increase the clinical efficacy of hormone therapy. Luque et al., 2021 showed that the CAFs, derived from HER2⁺ patients, promoted resistance to trastuzumab and pertuzumab treatment *in vitro*. However, the correlation with the presence and impact of CAF-S4 was not considered so far. The HER2-amplified breast cancer cells are resistant to tyrosine-kinase inhibitor lapatinib due to CAF manipulation of the ECM rigidity through YAP/TAZ activation (Lin et al., 2015). Soluble factors secreted by CAFs have been explained to cause tumor resistance to anti-HER2 therapies. CAF-derived FGF5 in breast cancer has caused resistance to HER2-targeted therapies through activation of the FGFR2 and c-Src downstream pathways (Fernández-Nogueira et al., 2020). Other secretory growth factors of interest including TGFβ and HGF regulate cancer-related pathways and tumor progression which can be tied to HER2 therapy resistance (Ikushima and Miyazono, 2010; Luraghi et al., 2014). Using a multi-omics approach to identify cytokines, transcription factors, kinases, and miRNAs or other secretory factors that CAFs release can aid in the identification of novel biomarkers that cause therapy resistance in HER2⁺ breast cancer and other forms of cancer.

### CAF-induced radiotherapy resistance

CAFs can influence the radioresistance of cancer cells with simultaneous activation of alternative mechanisms for their proliferation and progression. Irradiated fibroblasts have been observed to overcome apoptotic signaling and transform into activated phenotypes that promote tumorigenesis (Ansems and Span, 2020). In a recent study, co-cultured CAFs and cancer cells developed resistance to the clastogenic effects of 137Cs gamma rays due to the enhanced capacity of CAFs to repair DNA damage (Domogauer et al., 2021). It is likely that the CAFs that survived radiation therapy modulated the fate of associated cancer cells and caused radiation resistance in the tumor as a whole. Exosomes derived from colon CAFs contribute to radioresistance by promoting cancer stem-like phenotypes (Liu et al., 2020b). With CAF-derived exosomes comes the consequent activation of tumor-promoting signaling pathways and stemness induction, instilling resistance in tumor cells (Liu et al., 2020b). Production of TGFβ by radio-treated CAFs can also promote cancer cell aggressive and invasive properties, acting as a radiotherapy therapeutic escape and resistance (Papadopoulou and Kletsas, 2011; Zhang et al., 2017). Notch signaling in CAFs plays a role in radioresistance, as demonstrated by a recent study that identified single-cell transcriptomic profiles in CAF subpopulations in *delta-like canonical Notch ligand 1*, Dll1⁺ tumors (Nandi et al., 2022). The connection between Notch signaling and CAF-associated metastasis and radioresistance could be exploited to improve breast cancer patient outcomes by minimizing radioresistance and stemness (Nandi et al., 2022). The increased expression of CXCL12, TGFβ, MMPs, and HGF in CAFs induced by radiation increases the activation of EMT pathways in cancer cells (Ansems and Span, 2020). In the majority of radiotherapies, similar mechanisms involving CAF-derived ECM remodeling and desmoplasia, hypoxia-induced TME, autophagy-induced cancer cell recovery, secretory factors, exosomes, and miRNA cause downstream signaling of cyclization and tumor resistance (Mantoni et al., 2011; Horsman and Overgaard, 2016; Wang et al., 2017). Radiotherapy-induced immunomodulatory mechanisms include CAFs avoiding immune recognition and retaining immunosuppressive properties within the TME (Song et al., 2021). Overall, radiotherapy resistance observed in cancer cells has been linked to CAF activity in the tumor microenvironment; therefore, comprehensive studies on the effects of CAFs on radioresistance can lead to an increase in overall survival and the potential development of successful combination therapies.

### CAF and breast cancer immunotherapy

Recent research has shown that CAFs play a significant role in immunotherapy resistance by excluding T-cells from tumors. FAP⁺ CAFs can protect tumor cells from tumor necrosis factor and interferon-mediated T-cell necrosis and can express CXC motif chemokine ligands, inhibiting T-cells from accessing tumor cells. ECM remodeling by CAFs also creates a physical barrier preventing immune cell infiltration. The interplay between CAFs and tumor immunosuppressive cells is vital in discovering new ways to promote immunogenic cancer cell death. The degradation, suppression, or reduction of CAFs have been shown to reduce immunosuppressive cells and allow an increased T-cell mediated anti-tumor effect (Antonia et al., 2016). As previously described, the CAF-secretion of IL-6 and IL-8 can promote tumor growth. CAFs aggregate macrophages and activate the NF-κB pathway through the secretion of IL-6 and IL-8, resulting in tumor immunosuppression (Su et al., 2018).

In a scRNA-seq study of a subpopulation of CAFs called CAF-S1 from breast cancer, it was observed that by promoting the attraction, survival, and total quantity of CD4⁺/CD25⁺/FOXP3⁺ regulatory T-cells in the TME, these CAFs promote immunosuppression. More specifically associated with the immunosuppressive environment were the clusters ECM-myCAF and TGFβ-myCAF. The abundance of these clusters correlates with that of PD-1⁺ and CTLA4⁺ CD4⁺ T-cells. The expression of PD-1 and CTLA4 at the surface of FOXP3⁺ T regulatory cells is upregulated by the ECM-myCAFs. Conversely, CD4⁺ CD25⁺ T lymphocytes promote the transformation of ECM-myCAF into TGFβ-myCAF fibroblasts. All these findings allude to the crosstalk between specific CAF clusters and T-lymphocytes that plays a role in immunosuppression and immunotherapy resistance (Kieffer et al., 2020). CAFs also directly contribute to T-cell suppression and deactivation by emulating APCs. In this mechanism, the antigen complexed with MHC-I is processed and presented by these fibroblasts, followed by co-incident antigen-specific upregulation of PD-1/PD-L2 and FAS/FASL on CAFs and T-cells, respectively. The result is enhanced tumor viability due to the dysfunction and death of T-cells (Lakins et al., 2018b). Therefore, the relationship between the TME and breast cancer cells, which is intricately intertwined with the CAF and inflammatory cells, has enormous potential for advancements in the treatment of breast cancer.

A popular approach to targeting CAFs and their tumor-promoting effects includes CAF reprogramming or reversion to a quiescent phenotype. CAF-rich tumors exclude CD8^+^ T-cells at the tumor margin and upregulate the expression of CTLA-4. A result of this exclusion is the induction of resistance to various immunotherapies, even those that include therapeutic vaccination and αPD1. A study showed that myofibroblastic CAF differentiation is regulated by the enzyme NADPH oxidase-4 (NOX4), and inhibiting this enzyme can transform these myofibroblastic CAFs back to a quiescent, fibroblast-like phenotype (Hanley et al., 2017). Upon further examination using a small-molecule NOX4/1 inhibitor [GKT137831 (setanaxib), genyotex] to target this pathway, it was observed that these drugs could surpass CAF-mediated immunotherapy resistance. These drugs significantly increased CD8^+^ T-cell infiltration into tumors, efficiently reversed CAF differentiation *in vivo*, and enhanced the immunotherapy response (Hanley and Thomas, 2020). Other targeting strategies include targeting ECM to allow drug access to tumor cells, TGF-β1 inhibition to prevent tumor progression by facilitating T cell infiltration, and reprogramming CAFs back to a quiescent state by using vitamin A and D agonists to increase the efficacy of chemotherapy (Sherman et al., 2014; Shany et al., 2016; Liu et al., 2019b). Moreover, Hh inhibition remained a significant target in cancer types such as PDAC, where activated CAFs were seen to have elevated Hh signaling and corresponding iCAF production leading to more regulatory T-cells and increased immunosuppression (Steele et al., 2021). Other approaches involve the use of viral vaccines to broaden the cytotoxic range of therapy effects on tumor cells, and CAFs combined (Chen et al., 2015), CAF-directed drug conjugates, inhibition of CTLA-4 and PD-L1 stagnating tumor progression by increasing intratumoral CD8⁺and CD4⁺ T-cells while reducing T regulatory cell count and overall immunosuppressive actions (Fiegle et al., 2019), CXCL12 and CXCR4 inhibition of induced metastasis and invasiveness (Izumi et al., 2016), targeting CAF immune evasion mechanisms, and finally CAF depletion. In many cancers, TAM overexpresses CSF-1R receptors that control macrophages’ production, differentiation, and function. In a murine model, a CSF-1R inhibitor increased T-cell infiltration and CD8⁺ T-cell activity, reduced the recruitment of bone marrow-derived suppressor cells (BMDSC), and suppressed the generation of inflammatory mediators in TAM. As a result of the inhibition of CSF-1R, CAFs neutralized the inhibitor by secreting chemokines and chemokine ligands. The addition of a chemokine receptor antagonist decreased the tumor burden, and the addition of an immune checkpoint inhibitor (anti-PD-1) entirely stopped tumor growth (Kumar et al., 2017). Methods of targeting CAF populations related to immune modulation in the tumor microenvironment can prove to be effective in countering the effects of CAF-induced immunotherapy resistance.

## Targeting CAFs to prevent cancer progression and improve therapy efficacy

**FIGURE 4:**
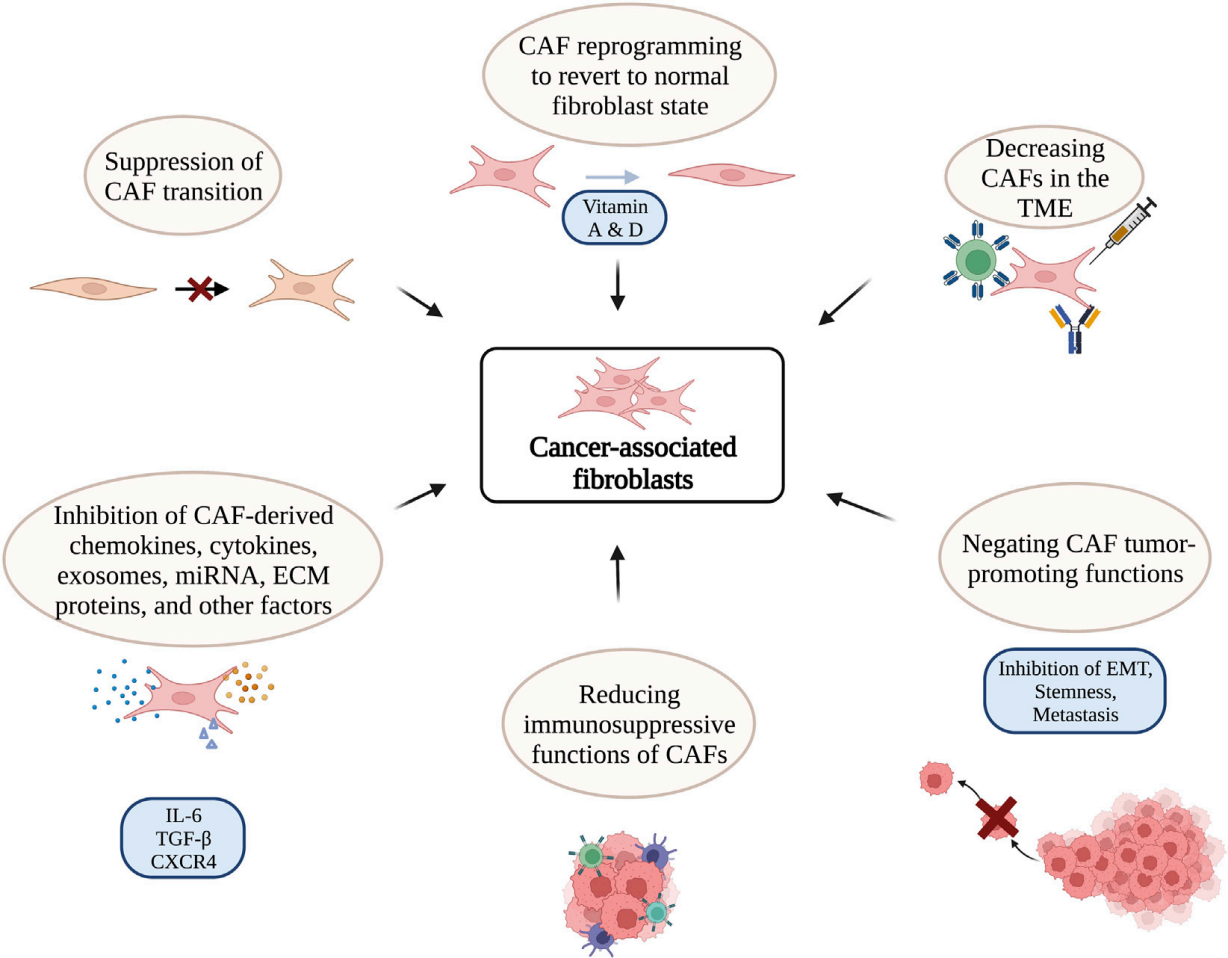
Various targeting strategies against CAFs^1^. This illustration includes CAF-targeting approaches, including suppression of CAF transition from inactive to active states (Hanley et al., 2017); CAF reprogramming by targeting Vitamin A&D receptors to revert to the quiescent state (Sherman et al., 2014; Ferrer-Mayorga et al., 2017); decreasing CAFs in TME through CAR-T-cell therapy (Sakemura et al., 2019), vaccination (Chen et al., 2015), monoclonal antibody (Hanley and Thomas, 2020); Negating CAF tumor-promoting functions through inhibition of EMT, stemness, and metastasis (Sakemura et al., 2019); reducing immunosuppressive functions of CAFs to achieve greater T-cell accessibility to tumor cells and increased sensitivity to therapeutic approaches (Hanley and Thomas, 2020); inhibition of CAF-derived chemokines, cytokines, exosomes, miRNA, ECM proteins, and other factors (Izumi et al., 2016).

The methods of anti-cancer treatment resistance induced by CAFs can be a gateway to targeting the innate function of CAFs and their effects on tumor progression and therapy efficacy. CAFs are influential in cancer progression and exhibit dichotomous effects of tumor inhibition in early stages and tumor promotion in advanced stages through CAF plasticity, transformation, and secreted growth factors and cytokines such as TGFβ or other secretory factors that induce tumor progression. Targeting specific populations of tumor-promoting CAFs can inhibit tumor progression and improve patient prognosis. Still, clinical studies have been unsuccessful due to the lack of specific biomarkers and their respective ability to transdifferentiate in response to various TME stimuli (Sanz-Moreno et al., 2011b; Özdemir et al., 2014; Sahai et al., 2020b). The different CAF-targeting strategies thus far (Figure 4) include– 1) suppression of CAF transition or formation of the activated state, 2) CAF reprogramming to revert to a NF state, 3) decreasing CAFs in the tumor microenvironment, 4) negating CAF tumor-promoting functions, 5) inhibition of CAF-derived factors, and 6) reducing immunosuppressive functions of CAFs. Using the described mechanisms of targeting CAFs along with immunotherapy, radiotherapy, chemotherapy, hormone therapy, or combined therapy, CAF-induced resistance to therapeutic approaches may be overcome.

Through a number of methods, including the exposure of neo-antigens, STING activation, and PD-L1 overexpression, radiation therapy has been demonstrated to synergize with immune modifying therapy (Zhang et al., 2008; Corso et al., 2011; Deng et al., 2014). Tumors can also transform after radiation therapy, making them more sensitive to immunotherapy (Demaria et al., 2005). Therefore, Combination therapy is another promising therapeutic strategy to combat tumor progression. However, the response to the combination of radiotherapy (RT) and immune treatment can vary. The challenge is to determine why some patients respond to RT and immune treatment with a long-lasting response and others with a limited response. CAFs enhance or reduce drug resistance depending on the context. Understanding the connection between the divergent CAF secretome contents and tumor signaling needs may help identify potent combination treatment approaches.

## Conclusion

In summary, CAFs play an essential role in the development and progression of breast cancer. In this review, we have summarized recent findings on the various potential origins and diverse functions of CAFs within the tumor microenvironment. These cells have been observed to be both tumor-promotive and tumor-suppressive depending on various factors found within and around the tumor microenvironment and on their heterogeneity. We discuss the molecular and functional heterogeneity of CAFs in order to demonstrate their involvement in different tumor-promoting mechanisms, such as the introduction of stemness, metastasis, migration, invasion, angiogenesis, and therapy resistance, *via* ECM remodeling, immunomodulation, and altered metabolic activities^1^. Several studies have been performed to classify different clusters and subsets of CAFs. New methods of classification such as scRNA seq have revealed several unique CAF subtypes and possible markers. A deeper understanding of the molecular and phenotypical differences between CAF populations can lead to addressing issues in therapy resistance and cancer cell targeting. CAF-induced resistance to therapy has been observed to contribute vastly to tumor progression and decreased patient survival. The precise approach of CAF-based treatment is significantly impeded by the fact that the CAFs do not express αSMA or FAP, or any other cell surface marker exclusively. That underscores the need for more knowledge on the numerous strategies CAFs employ to enhance cancer development in the backdrop of a range of therapeutic interventions. This can aid in developing optimal treatment plans and sustained therapy efficacy.

Therefore, from helping in angiogenesis and metastatic invasion to maintaining stemness, CAFs have been established as a potential therapeutic target in breast cancer. Although several exciting and critical findings have been reported in recent years, studies should focus on comparing CAF populations 1) in multiple tumors, 2) in different stages of malignancies, 3) in functional characterization of distinct CAF subtypes, and 4) in development of subtype-specific therapies. Together, this will help to build a more comprehensive picture of the relationship between CAFs, tumor microenvironment, and therapy response.

## Footnotes

1 Created with BioRender.com.
